# Non-locked and locked small fragment straight plates have a similar behavior in buttressing the posteromedial shear tibial plateau fragment: a biomechanical analysis of three different fixations

**DOI:** 10.1186/s40634-020-0218-0

**Published:** 2020-01-17

**Authors:** Vincenzo Giordano, Mauricio Kfuri, William Belangero, Allison Venturini, Ana Carolina Silva, Eduardo Merjan Soares, Robinson Esteves Pires, Hilton A. Koch

**Affiliations:** 1 Serviço de Ortopedia e Traumatologia Prof. Nova Monteiro, Hospital Municipal Miguel Couto, Rio de Janeiro, RJ Brazil; 2 Clínica São Vicente, Rede D’or São Luiz, Rua Mário Ribeiro 117/2° andar, Leblon, Rio de Janeiro, RJ 22430-160 Brazil; 3 Department of Orthopedic Surgery, University of Missouri, Columbia, MO USA; 4 Departamento de Biomecânica, Medicina e Reabilitação do Sistema Locomotor, Escola de Medicina de Ribeirão Preto, Universidade de São Paulo (USP), Ribeirão Preto, SP Brazil; 5 Laboratório de Biomateriais em Ortopedia, Faculdade de Ciências Médicas, Universidade de Campinas (UNICAMP), Campinas, SP Brazil; 6 Departamento de Ortopedia, Faculdade de Ciências Médicas, Universidade de Campinas (UNICAMP), Campinas, SP Brazil; 7 Departamento de Ortopedia, Universidade Federal de Minas Gerais (UFMG), Belo Horizonte, MG Brazil; 8 Departamento de Radiologia, Universidade Federal do Rio de Janeiro (UFRJ), Rio de Janeiro, RJ Brazil

**Keywords:** Tibial plateau fracture, Proximal tibia fracture, Posteromedial fragment, Biomechanical study

## Abstract

**Purpose:**

The aim of this study is to compare the biomechanical behavior of three different fixation constructions currently used for buttressing the posteromedial shearing tibial plateau fragment. Our hypothesis is that non-locked implants provide sufficient comparable stability in posteromedial tibial plateau fractures as locked implants.

**Methods:**

Fifteen left synthetic tibiae from a single manufacturing batch were used to create a posteromedial shear tibial plateau fracture. The fracture was buttressed with three different posteriorly placed five-hole straight small-fragment plate. Five models were fixed with a one-third tubular plate (TTP), five models with a dynamic compression plate (DCP), and five models with a locking compression plate (LCP). All groups were tested to vertical subsidence (Stage 1). In the same experiment (Stage 2), TTP and DCP groups were tested until catastrophic failure. Force versus displacement curves were obtained in the two stages of the experiment.

**Results:**

Stage 1 – There was no significant difference in stiffness (*p* = 0.89), subsidence up to 2 mm (*p* = 0.38), and energy (*p* = 0.36) among the three fixation constructions. Stage 2 – Yield load revealed significantly less yield strength for the TTP group as compared with the DCP group (*p* = 0.048). However, there was no significant difference in maximum load to failure among the TTP and DCP fixation constructions (*p* = 0.16).

**Conclusion:**

Placement of either a locked or non-locked small fragment straight plate to buttress the posteromedial shear tibial plateau fragment has a similar biomechanical behavior. When the implant is positioned to buttress the shearing fragment it maximizes biomechanical stiffness.

## Introduction

Posteromedial fracture of the tibial plateau is a relatively common but overlooked injury, with devastating consequences in knee function and stability [1, 2]. The fracture mechanism is a combination of axial compression and varus stress with the knee in a semi-flexed position, producing an often grossly displaced condylar split fracture of tibial plateau oriented in the coronal plane [1–3]. Anatomical reduction of the articular surface, restoration of the normal posterior slope of the tibial plateau, and management of the posteromedial corner capsule-ligamentous structures are critical for a painless stable knee joint [4, 5].

Normally a posteromedially-based approach with direct reduction and rigid fixation using a buttress plate is considered a standard of care to treat an isolated posteromedial tibial plateau fracture [6, 7]. The optimal placement of the plate should be parallel to the main fracture plane, as described by Kfuri and Schatzker [8]. However, the choice of approach (either Tscherne-Lobenhoffer or Luo), position of the patient, and type of implant are all variables determined by the surgeon based mainly on the fracture pattern and personal experience and confidence. In a biomechanical in-vitro strength analysis of four different fixation methods, Zeng et al. have shown that a posterior-based buttress technique using a 3.5-mm six-hole T-shaped plate is biomechanically superior than the other methods [7]. Although not pointed out as a limitation, the authors tested only one fracture model using a posterior buttress plate construction.

To the best of our knowledge, a comparative evaluation between locked and non-locked small fragment straight implants used to buttress the posteromedial shear tibial plateau fragment was not investigated so far. Locking plates were primarily designed to address fragility fractures or high energy periarticular fractures. Despite all theoretical mechanical advantages of these implants, the question is whether they are absolutely needed for the fixation of posteromedial split wedge fractures. The aim of this study is to compare the biomechanical behavior of three different fixation constructions currently used for buttressing the posteromedial shearing tibial plateau fragment. Our hypothesis is that provided the plate is placed parallel to the main fracture plane, locking constructs have no mechanical advantage over conventional ones.

## Methods

### Posteromedial fracture preparation

Fifteen left synthetic tibiae (Model 1110, Synbone AG, Swiss) from a single manufacturing batch were used to create a posteromedial shear tibial plateau fracture.

**Fig. 1:**
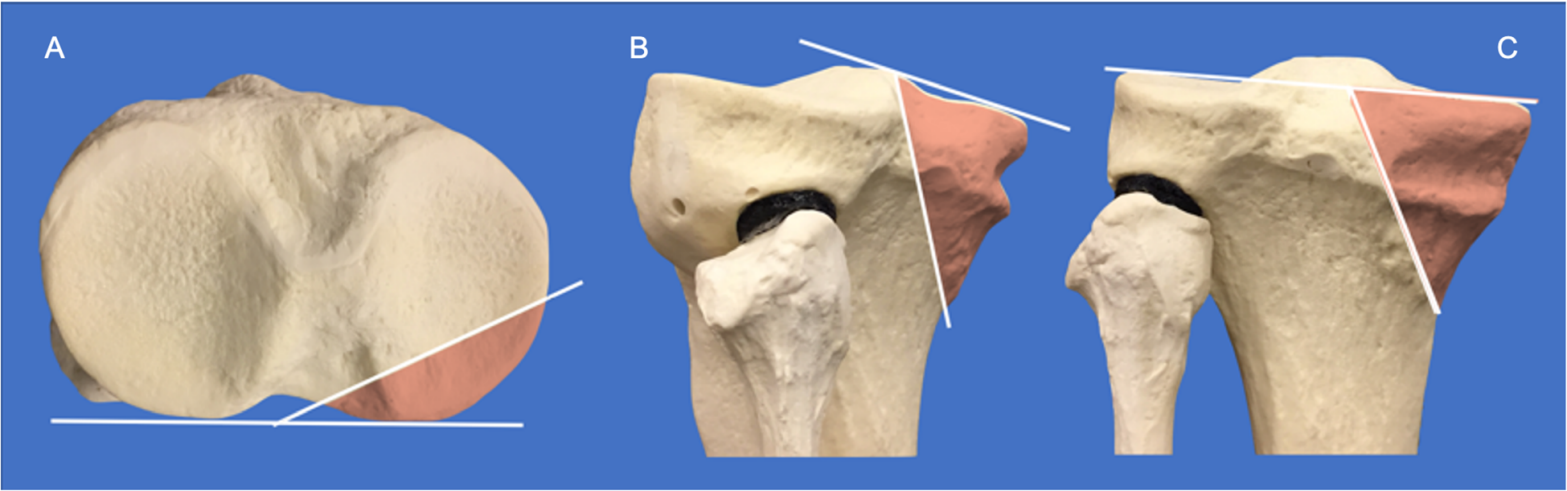
A posteromedial fracture line was drawn with the articular fragment angle (α) of approximately −25° and the sagittal angle (β) equal to 75°. Note the orientation of the fracture on the axial view (**a**), internal oblique view (**b**), and coronal posterior view (**c**)

A fracture line was draw based on the model designed for Zeng et al. [7]. The cut was done with a band saw starting from the articular surface and exiting the posterior side of the synthetic model, making a sagittal angle of 75° (Fig. 1).

The fracture was directly reduced with a large pointed reduction clamp and fixed with three different posteriorly placed five-hole buttress plate. Five models were fixed with a one-third tubular plate (TTP), five models were fixed with a small fragment dynamic compression plate (DCP), and five models were fixed with a small fragment locking compression plate (LCP). The non-locked plates were from Ortosintese (Jaraguá, Brazil) and the locked plate was from DPS (Paoli, USA). The implants from both DCP and LCP groups were bent to fit the contour of the posteromedial tibial plateau; this was not done for the TTP group.

**Fig. 2:**
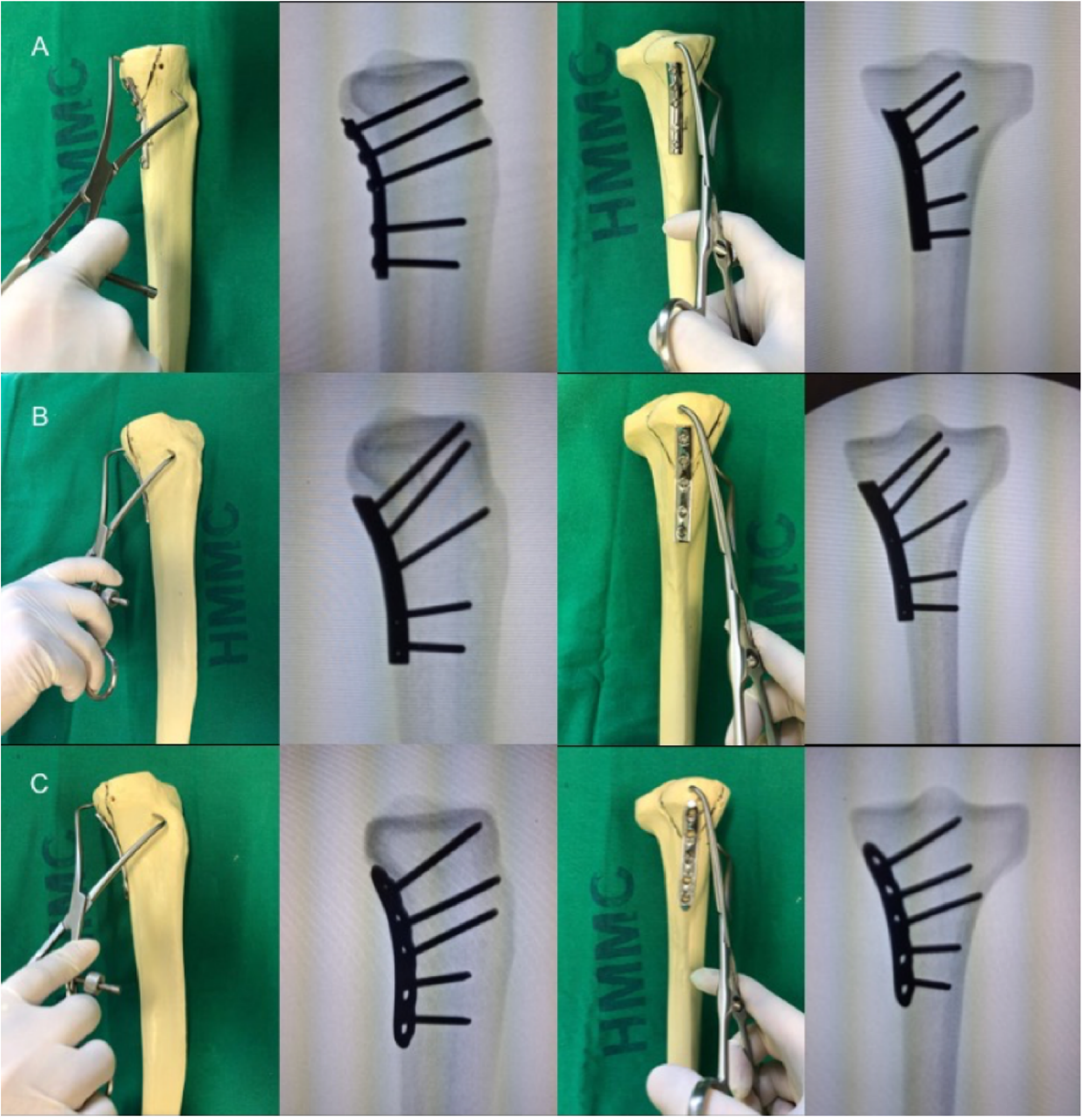
**a** TTP specimen model, **b** DCP specimen model, and **c** LCP specimen model. Note the perfect contouring of the plates to the posteromedial surface of the tibial plateau. Anatomic reduction was warranted both by direct vision and fluoroscopic control

The first screw in all experimental groups was a non-locked cortical screw inserted 1-mm distal to the apex of the triangular fragment. The other screws were inserted in an alternate manner starting from the second more distal screw-hole. In the LCP group except form the first screw all other screws were locked. In all groups, screws were bicortical and directed for the anterior cortex. Anteroposterior (AP) and lateral fluoroscopic images were obtained for each tibial plateau-implant construct to check for any incongruency on the position of the screws (Fig. 2).

The models were sawed with a length of 170 mm for adjustment to the biomechanical testing machine.

### Biomechanical testing

The tests were performed on an MTS 810 material testing system Model 318.10 with a maximum force capacity of 100 kN integrated to a FlexTest 40 digital servocontroller (MTS Systems Corporation, Eden Prairie, USA). A load cell with a capacity of 10 kN was used in the tests. The experiment was held on the Mechanical Testing Laboratory, Department of Manufacturing and Materials Engineering, Faculty of Mechanical Engineering – UNICAMP (Campinas, Brazil).

**Fig. 3:**
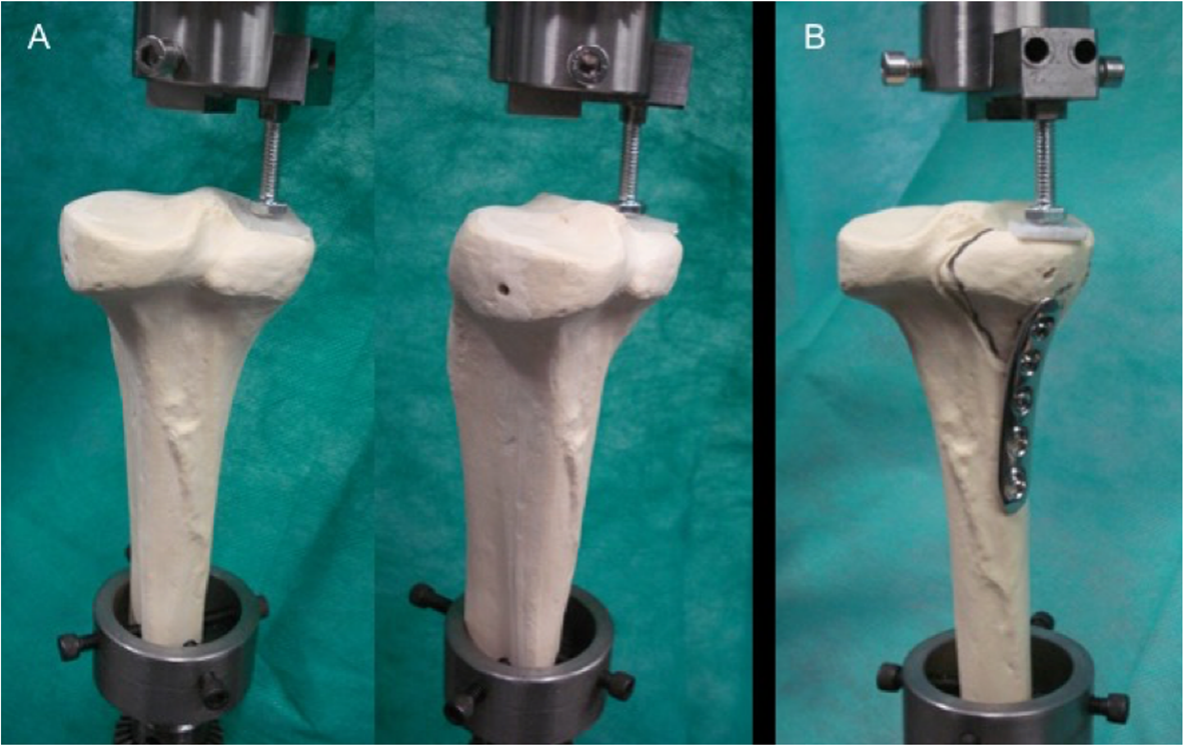
**a** The specimens were positioned vertically onto a test jig with a polypropylene plate measuring 20 × 15 × 3 mm clamped on the upper side of the material testing machine to deliver forces at the point of loading on the posteromedial tibial plateau. **b** LCP specimen model before testing

The specimens were positioned vertically onto a test jig. A polypropylene plate measuring 20 × 15 × 3 mm was clamped on the upper side of the material testing machine and used as an applicator to deliver forces at the point of loading on the posteromedial tibial plateau (Fig. 3) [9]. A vertical pre-load of 40 N was applied before the tests were carried out. The vertical load was applied to generate axial compression with a loading speed of 1 mm/minute.

**Fig. 4:**
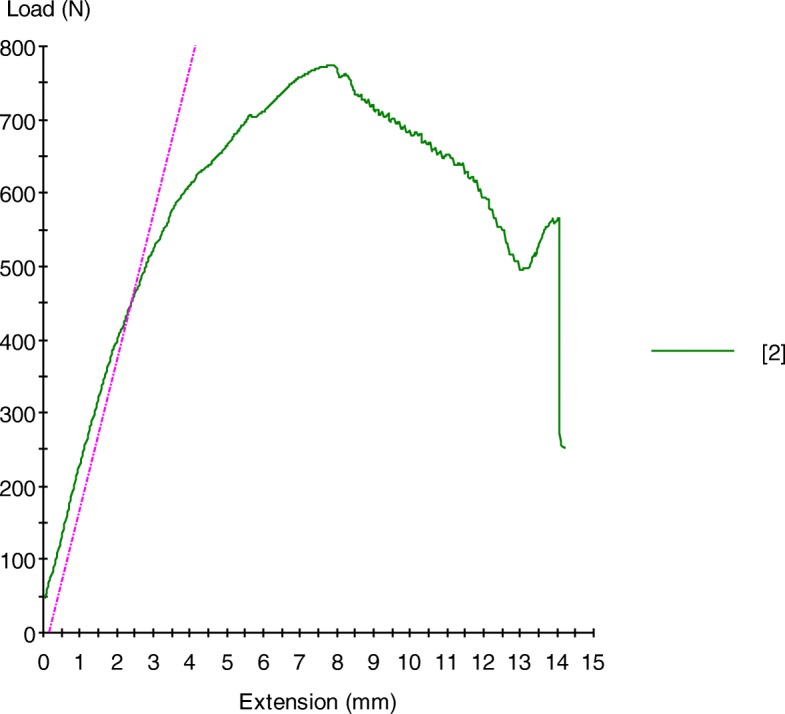
Yield strength (green) vs ultimate strength (pink) of a specimen of DCP group at Stage 2. Yield load was determined for the TTP and DCP groups at the point where the curve ceased to be linear and suffered an inflection. The yield point was determined when there was a crossing between the dashed pink line and the green curve

All groups were tested up to 2 mm displacement defined as subsidence of the posteromedial fragment (Stage 1). For the TTP and DCP groups the specimens were tested until failure load defined as bone-plate construction failure or catastrophic failure (Stage 2). Yield load was determined for the TTP and DCP groups at the point where the curve ceased to be linear and suffered an inflection (Fig. 4). Force versus displacement curves were obtained in the two stages of the experiment.

### Statistical analysis

All statistical calculations were carried out using SPSS Version 20.0 (SPSS Inc., Chicago, USA). Previous to the beginning of the experiment, the size of the sample was calculated by using the type-II error (beta-error analysis) for the student’s t test, with a Cohen’s d effect size of 0.2, founding a beta level (two-tailed hypothesis) of 0.967. This was considered adequate in terms of the number of plastic bone models used.

Descriptive statistics was used to determine ranges, means, and standard deviations.

Statistical analysis was performed using one-way analysis of variance (ANOVA one-way) and student’s t-test for independent samples to compare stiffness (N / mm), maximum load up to 2 mm (N), energy (J), yield load (N), and maximum load to failure (N) between construction groups [10]. A *p* value of < 0.05 was set as the level of significance. Inferential analysis was composed by ANOVA one-way in order to compare stiffness (N / mm), maximum load up to 2 mm (N), and energy (J) between construction groups, and student’s t-test for independent samples to compare yield load (N) and maximum load to failure (N) between TTP and DCP groups.

Normality test of Shapiro-Wilk and graphical histogram analysis were used to compare the scores in the sample to a normally distributed set of scores with the same mean and standard deviation [11]. Data presented a Gaussian distribution by not rejecting the null hypothesis, assuming that the samples were normally distributed.

## Results

### Stage 1 – subsidence of the posteromedial fragment

**Fig. 5:**
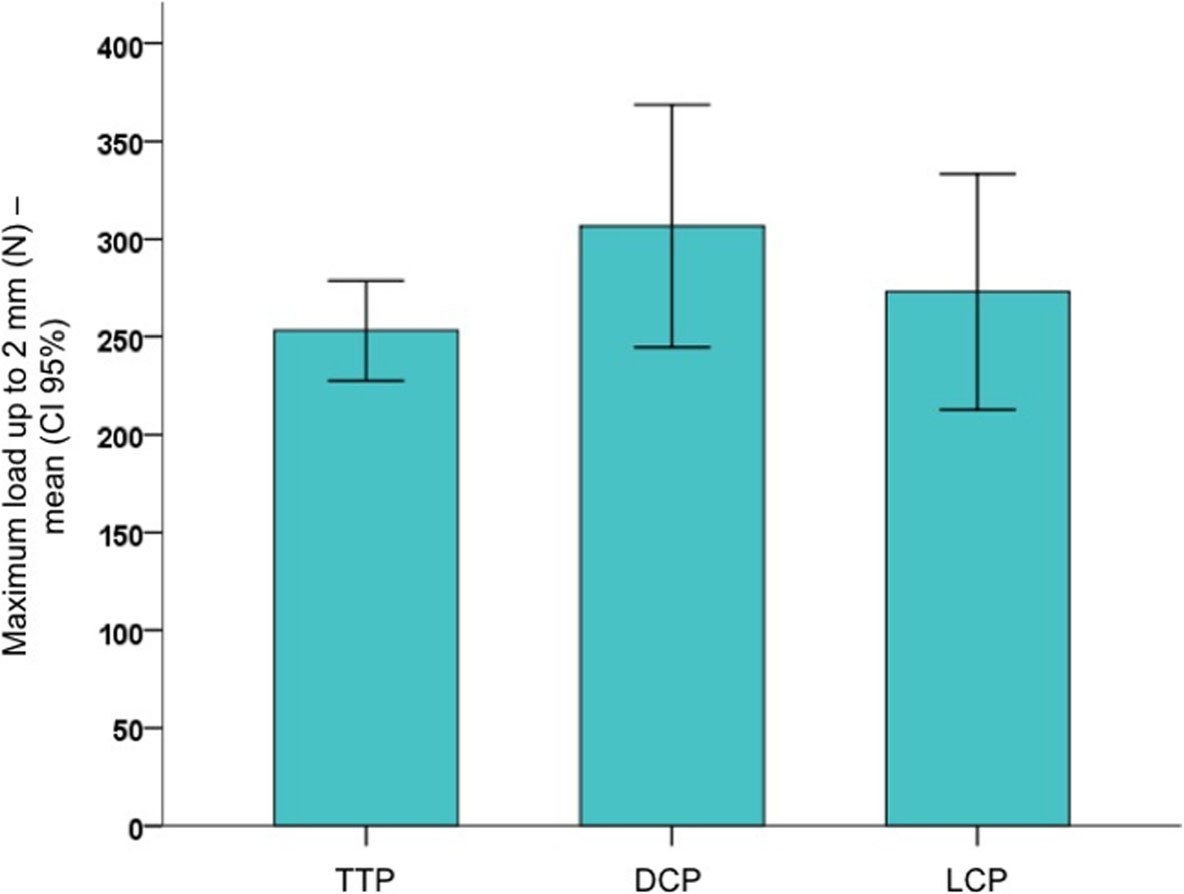
Maximum load up to 2 mm (subsidence) for the three experimental groups (mean and CI 95%)

There was no significant difference in stiffness (*p* = 0.89), maximum load up to 2 mm (*p* = 0.38), and energy (*p* = 0.36) among the three fixation constructions. Mean stiffnesses were 177 (CI 159–195) N/mm for TTP group, 188 (CI 168–208) N/mm for DCP group, and 183 (CI 150–215) N/mm for LCP group. Mean maximum load were 253 (CI 228–279) N for TTP group, 307 (CI 245–369) N for DCP group, and 273 (CI 213–333) N for LCP group (Fig. 5). Mean energy was 0.25 (CI 0.23–0.28) J for TTP group, 0.31 (CI 0.24–0.37) J for DCP group, and 0.28 (CI 0.22–0.34) J for LCP group. Data is summarized in Table 1.

**Table 1:** Subsidence of the posteromedial fragment up to 2 mm (Stage 1 – TTP vs DCP vs LCP group)

Parameter / group	n	mean	CI 95%			minimum	maximum	*p value* ^*a*^
Stiffness (N/mm)								
TTP	5	177	159	–	195	155	200	0.82
DCP	5	188	168	–	208	170	224	
LCP	5	183	150	–	215	146	241	
Maximum load (N)								
TTP	5	253	228	–	279	223	301	0.38
DCP	5	307	245	–	369	211	398	
LCP	5	273	213	–	333	207	377	
Energy (J)								
TTP	5	0.25	0.23	–	0.28	0.22	0.30	0.36
DCP	5	0.31	0.24	–	0.37	0.21	0.40	
LCP	5	0.28	0.22	–	0.34	0.21	0.38	

^a^ANOVA one-way

### Stage 2 – catastrophic failure

**Fig. 6:**
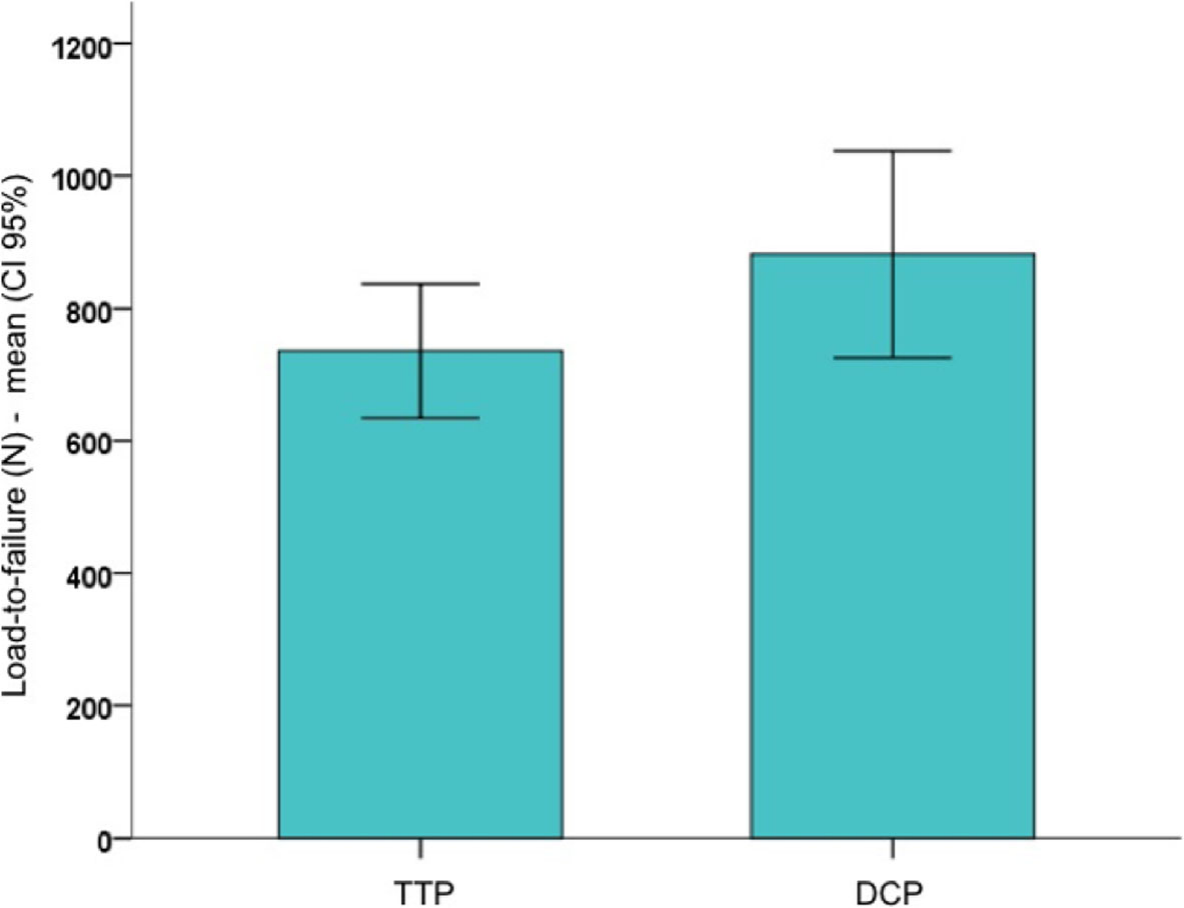
Load-to-failure for the TTP and DCP experimental groups (mean and CI 95%)

Yield load revealed significantly less yield strength for the TTP group as compared with the DCP group (*p* = 0.048). Mean yield load was 632 (CI 572–691) N for TTP group and 790 (CI 671–909) N for DCP group. There was no significant difference in maximum load to failure among the TTP and DCP fixation constructions (*p* = 0.16). Mean failure load was 736 (CI 634–838) N for TTP group and 882 (CI 735–1038) N for DCP group (Fig. 6). Data is summarized in Table 2.

**Table 2:** Catastrophic failure (Stage 2 – TTP vs DCP group)

Parameter / group	n	mean	CI 95%			minimum	maximum	*p value* ^*a*^
Yield load (N)								
TTP	5	**632**	572	–	691	569	732	**0.048**
DCP	5	**790**	671	–	909	577	944	
Load-to-failure (N)								
TTP	5	736	634	–	838	606	875	0.16
DCP	5	882	725	–	1038	612	1113	

^a^Student t test for independent samples

01.006

**Fig. 7:**
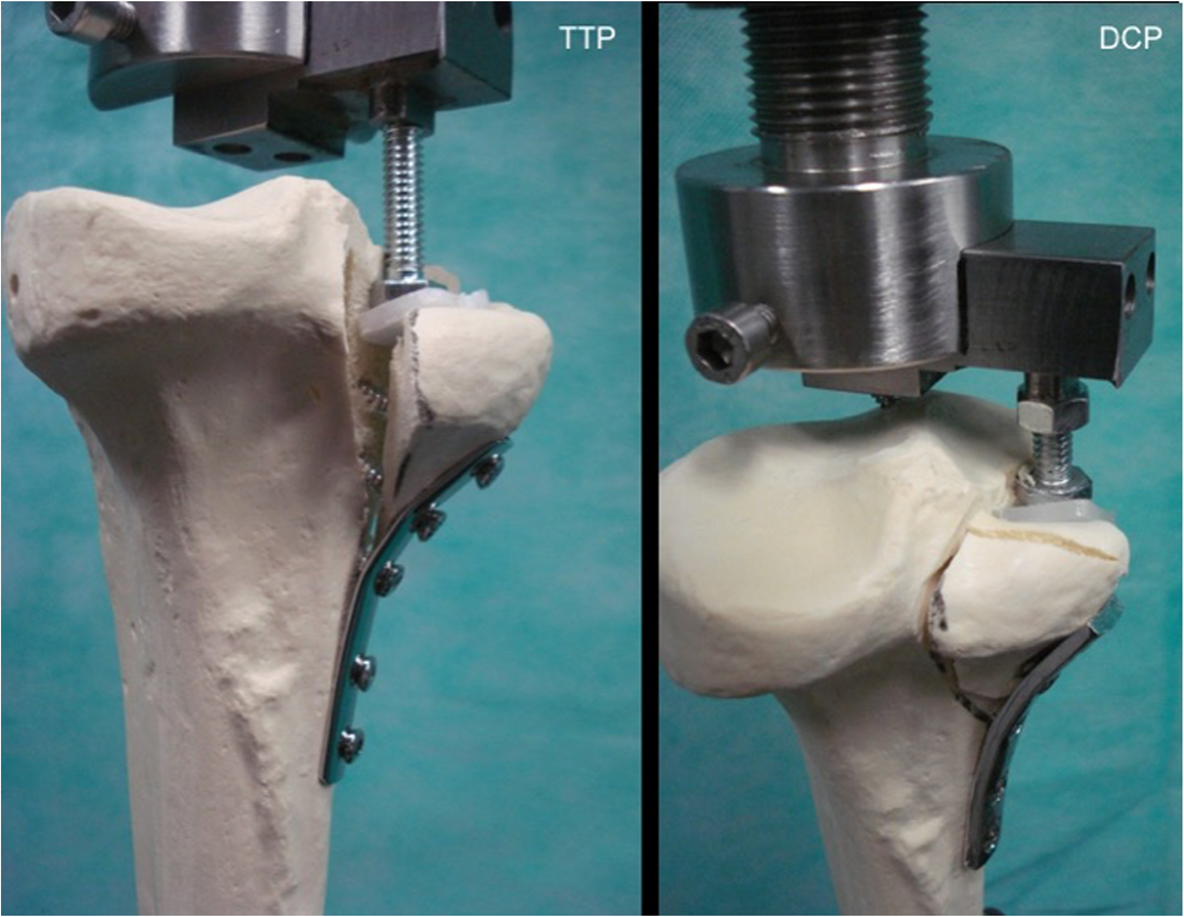
Mode of failure for the TTP and DCP constructs, represented by one specimen of each experimental group

All specimens in the TTP group failed by a combination of overbending of the plate and proximal screws pull-out, whereas in DCP group specimens failed by fragmentation of the posteromedial tibial plateau fracture propagated through the proximal screw shafts. The failure mode of constructs is shown in Fig. 7.

## Discussion

The main findings of the present biomechanical study are that there is no significant difference in stiffness, subsidence up to 2 mm, and energy between non-locked and locked fixation constructions tested for buttressing the posteromedial shearing tibial plateau fragment. Although not reached statistical significance, despite an adequate number of plastic bone models used, as calculated previously to the beginning of the experiment, specimens from the DCP group showed the stiffest bone-implant construction under axial loading, which may result from bigger cross-sectional area of the implant compared to the one-third tubular plate and the locking compression plate. TTP and DCP groups were further tested until catastrophic failure. Yield load revealed significantly less yield strength for the TTP group as compared with the DCP group. Y*ield* strength is a material property defined as the stress at which a material begins to deform plastically, suffering permanent irreversible damage. The yield strength would correspond to the yield load divided by the area of the specimen. Since we cannot define the area, we cannot determine this property. Therefore, instead of presenting the results in terms of strength (MPa), we present them in terms of load (N).

A previous biomechanical study on four different fixation methods for a posteromedial tibial plateau split fracture indicated that a posterior small fragment locked T-shaped buttress plate produced significantly greater stability in controlling the subsidence of the posteromedial fragment under axial loading [7]. Zeng et al. have argued that osteopenia and fracture comminution should potentially affect the stability provided by non-locked plates, which rely on screw–bone interface. It should be noted, however, that a significant proportion of patients that suffer a posteromedial tibial plateau fracture are young and middle-aged male, with approximately one fifth presenting comminution of the articular surface [1, 2]. Moreover, as mentioned before the authors tested only one fracture model using a posterior buttress plate construction [7].

Few studies have specific examined the posteromedial fracture of the tibial plateau, either as an isolated injury or associated with other proximal tibia fracture. Some authors have found this pattern in approximately 18% of isolated fractures and in near one third of bicondylar tibial plateau fractures [1, 3]. Hohl was the first to describe this injury as a split fracture of the posterior margin of the medial tibial plateau [12]. More recently some authors have evaluated the morphologic characteristics of the posteromedial fragment in bycondilar injuries [1, 2]. Barei et al. observed an averaged compromise of approximately 58% of the articular surface of the medial tibial plateau with a mean posteromedial fragment height of 4.2 cm and a mean sagittal fracture angle of 81° [1]. Higgins et al. noted more than 5 mm of articular displacement in 55% of cases with the posteromedial fragment exhibiting a vertical fracture pattern averaging 73° sagittal angle, highly indicative of shear instability and vertical displacement [2].

There is a general consensus that the posteromedial fragment should be directly reduced through a posteromedially based approach and fixed with a buttress plate placed to the apex of the triangular fragment [5, 8]. However, the definition of which plate should be used continues a matter of debate with some authors reporting the placement of different plates in the tibial plateau for buttressing the posteromedial fragment. De Boeck and Opdecam used a posterior large fragment T-shaped anti-glide plate for seven patients, with excellent and good results and no complications [13]. Bhattacharyya et al. reported on 13 patients with posterior shearing tibial plateau fractures treated through a posterior approach to the knee fixed with a 3.5-mm cloverleaf plate and found eight satisfactory results of nine responding patients [14]. Brunner et al. presented a series of five patients treated by open reduction and internal fixation with a dorsal 3.5 mm anti-gliding plate as a buttress plate with all patients highly satisfied with the postoperative result [15].

The present biomechanical study proves that there is no significant difference in stiffness, subsidence up to 2 mm, and energy between non-locked and locked fixation constructions. Although not reached statistical significance, specimens from the DCP group showed the stiffest bone-implant construction under axial loading, which may result from bigger cross-sectional area of the implant compared to the one-third tubular plate and the locking compression plate. TTP and DCP groups were further tested until catastrophic failure. Yield load revealed significantly less yield strength for the TTP group as compared with the DCP group. Y*ield* strength is a material property defined as the stress at which a material begins to deform plastically, suffering permanent irreversible damage. The yield strength would correspond to the yield load divided by the area of the specimen. Since we cannot define the area, we cannot determine this property. Therefore, instead of presenting the results in terms of strength (MPa), we present them in terms of load (N).

Our results show both that subsidence up to 2 mm and catastrophic failure are not influenced by the implant, either locked or non-locked, resulting in a relatively similar load transfer at the bone-plate interface. Previous studies demonstrated the importance of buttressing the medial tibial plateau split fragment with a plate, either isolated or associated to a lateral tibial plateau fracture [7, 9, 16]. It is suggested that the decreased stress on the medial proximal triangular fragment is likely the consequence of better anchorage and load sharing provided by perfect contouring of the plate [16]. In order to obtain maximum bone-plate interface, we suggest that the first screw inserted should be a non-locked cortical screw, approximately 1-mm distal to the apex of the triangular fragment.

There are few limitations of the present study. First, synthetic tibiae may not reflect the actual conditions of bone properties. However, similar biomechanical studies have demonstrated good reproducibility using plastic bones to evaluate different assemblies for fixation of medial tibial plateau fractures [7, 9]. Furthermore, it has been showed that plastic bone models present an advantage over fresh or frozen human bones because the setup variability of natural tibia axial stiffness is unacceptably high, indicating exceptional difficulty in obtaining reproducible bone alignment [2, 17–19]. This, the use of composite tibiae allows small differences to be characterized significantly, even when a small sample is used. Secondly, the synthetic models used reproduced the properties of a normal rather than osteoporotic bone. Therefore, our findings cannot be extrapolated to a clinical situation of poor bone stock. It has been suggested that locking plates appeared less sensitive to bone quality as they rely on the screw-plate interface [7]. Thirdly, we didn’t evaluate the catastrophic failure for the LCP group. This happened due to limited financial resources to obtain a similar number of locked plates to evaluate this group on the second phase of the experiment. Nevertheless, the number of plastic bone models used were considered adequate. In addition, for consistency, due to the reduced sample size, the Kruskal-Wallis ANOVA nonparametric test was applied, which corroborated the conclusions reached by the parametric approach (this analysis was performed for verification purposes only). Finally, stiffness, subsidence, and energy for all three groups and catastrophic failure between non-locked constructions were statistically similar. It can be hypothesized that our findings were underpowered to determine a difference in this experiment and that more specimens would be required to detect a significant difference between groups. It should be noted however, that the differences between data for all variables studied were small and therefore unlikely to be relevant in a statistical scenario.

In conclusion, this study suggests that placement of either a locked or non-locked small fragment straight plate to buttress the posteromedial shear tibial plateau fragment has a similar biomechanical behavior. When the implant is positioned to buttress the shearing fragment it maximizes biomechanical stiffness.
